# The Association Between Parental Homework Checking and Chinese Adolescents’ Loneliness: The Mediating Role of Academic Pressure and the Moderating Role of Parental Educational Expectations

**DOI:** 10.3390/bs16060860

**Published:** 2026-05-27

**Authors:** Wenbin Wu, Mingzheng Liu

**Affiliations:** School of Journalism and Communication, Jilin University, Changchun 130012, China; wuwb22@mails.jlu.edu.cn

**Keywords:** parental homework checking, academic pressure, loneliness, parental educational expectations, adolescent mental health

## Abstract

Driven by the Confucian cultural ideal of “wang zi cheng long”—the fervent hope that one’s child will rise like a dragon (i.e., achieve extraordinary success)—Chinese parents commonly engage in intensive academic involvement, such as frequent homework checking. However, the mechanisms through which this high-intensity monitoring affects adolescent mental health, and whether its effects are culturally specific, remain underexplored. Drawing upon the stimulus–organism–response (SOR) theory and the stress process model, this study used data from the 2022 China Family Panel Studies (CFPS) on 1831 adolescents aged 9–15 to examine the impact of parental homework checking frequency on adolescent loneliness, the mediating role of academic pressure, and the moderating role of parental educational expectations. The results show that parental homework checking frequency was positively associated with academic pressure, which in turn was positively associated with loneliness. The mediating role of academic pressure was significant. Parental educational expectations significantly and negatively moderated the relationship between homework checking and academic pressure, and the moderated mediation was significant. Simple slope analysis indicated that the positive association between homework checking and academic pressure was stronger. In the Confucian cultural context that emphasizes academic achievement and filial responsibility, frequent parental homework checking is associated with adolescent loneliness through increased academic pressure. Unexpectedly, high parental expectations served as a buffer—a pattern that differs from typical findings in Western individualistic cultures, where high expectations often directly increase psychological distress. These findings suggest that interventions in Chinese family education should distinguish controlling from supportive monitoring and transform high expectations into emotional support and resource investment, thereby reducing adolescents’ academic pressure and loneliness.

## 1. Introduction

Adolescent mental health has become a global public health priority. The World Health Organization estimates that approximately 10–20% of adolescents aged 10–19 worldwide suffer from some form of mental disorder, among which loneliness is particularly prevalent during adolescence and has profound long-term consequences. Chronic loneliness is not only closely associated with depression, anxiety, and suicidal ideation but also impairs academic performance, social competence, and adult mental health (Qualter et al., 2015; Loades et al., 2020). In China, with rapid socioeconomic transformation and increasingly intense competition in basic education, adolescent mental health problems are on the rise. The Report on National Mental Health Development in China (Fu & Zhang, 2021) indicated that about 24.6% of adolescents experience mild to severe depressive symptoms, with loneliness being one of the most commonly reported emotional experiences.

Chinese adolescents face more pronounced educational pressures than their Western counterparts—a difference with deep cultural roots. For thousands of years, the Confucian ideal that “a successful scholar can become an official” has profoundly shaped Chinese families’ educational values—education is regarded as the primary pathway for social mobility, and academic achievement is an important way to bring honor to the family. Under this cultural tradition, parents internalize high educational expectations as a family responsibility, making academic concern a core element of parenting practices (Hung, 2018). From the perspective of Hofstede’s Cultural Dimensions Theory, Chinese culture scores extremely high on Long-Term Orientation, emphasizing sustained investment in the future, delayed gratification, and perseverance (Hofstede & Minkov, 2010). Compared with short-term oriented Western cultures, this long-term orientation predisposes Chinese parents to make long-term, high-intensity educational investments in their children, thereby further reinforcing the emphasis on academic achievement and high expectations. Existing research has shown that students in East Asian Confucian heritage cultures generally experience higher academic pressure and that their parents’ educational expectations are significantly higher than those in Western countries (Steare et al., 2023). Therefore, understanding the issue of educational pressure among Chinese adolescents requires a thorough examination of its underlying cultural drivers.

Under the traditional East Asian value of “wang zi cheng long, wang nü cheng feng” (hoping that one’s son will become a dragon and one’s daughter a phoenix—i.e., achieving extraordinary success), Chinese parents commonly engage in deep academic involvement, including checking homework, arranging tutoring, and communicating with teachers. This “educational involvement” is thought to improve academic performance, but it may also translate into adolescents’ academic pressure, thereby being linked to emotional distress and loneliness. The existing literature suggests that parental educational involvement has a double-edged sword effect on adolescent mental health, but it is crucial to distinguish between controlling monitoring and supportive monitoring. Controlling monitoring is characterized by frequent checking, demanding expectations, and a lack of emotional warmth; it signals “your performance needs to be monitored” and tends to undermine adolescents’ sense of autonomy and competence, thereby increasing pressure and distress (Bradshaw et al., 2024). In contrast, supportive monitoring accompanies homework checking with encouraging language, resource investment, and respect for the child’s autonomy, which can satisfy basic psychological needs and buffer against negative emotions (Tanaka et al., 2023). On the one hand, moderate supervision and support (supportive monitoring) can enhance academic achievement and self-efficacy (G. Li et al., 2023; Cui & Liu, 2025). On the other hand, excessive monitoring, intrusive involvement, and high academic demands (controlling monitoring) can induce anxiety, depression, and social withdrawal (Y. Ma et al., 2018; L. Ma et al., 2021). However, most studies have focused on parental educational expectations or general educational support, paying insufficient attention to specific, high-frequency monitoring behaviors such as homework checking. Homework checking is a typical, observable parental behavior that, when performed frequently and without emotional support, aligns closely with the concept of controlling monitoring. Unlike educational expectations, homework checking is direct, frequent, and intrusive in daily interactions, and its predictive role on psychological pressure may be more immediate. Therefore, it is necessary to shift the research perspective from “attitudes” to “behaviors” and investigate the independent impact of the frequency of parental homework checking on adolescent mental health. We acknowledge that our data do not directly measure the emotional tone or quality of homework checking; thus, we interpret frequency as an indicator of controlling monitoring, and we discuss this limitation in Section 5.4.

To explain these mechanisms, the present study draws on the stimulus–organism–response (SOR) theory (Mehrabian & Russell, 1974) and the stress process model (Pearlin et al., 1981). The SOR theory posits that environmental characteristics serve as stimuli (S), triggering an individual’s internal psychological state (O), which ultimately is associated with to behavioral or health responses (R). The stress process model emphasizes that stressors ultimately affect mental health outcomes through individuals’ perceptions of stress. Based on these frameworks, this study proposes a mediation model: parental homework checking frequency is related to adolescent loneliness through academic pressure. Furthermore, parental educational expectations, as an important family background factor, may moderate the above path—parents with high expectations may be more inclined to adopt supportive monitoring, thereby weakening the pressure-related association of homework checking, whereas low expectations may amplify the pressure. Accordingly, this study further examines the moderating role of parental educational expectations.

The theoretical contributions of this study are threefold. First, it integrates SOR theory and the stress process model into the domain of family education, validating the chain of “external stimulus → cognitive appraisal → health outcome.” Second, it distinguishes between the “behavioral” and “expectational” dimensions of parental educational involvement, focusing on a specific monitoring behavior (homework checking frequency) and revealing its psychological costs independent of attitudes, while also uncovering the interaction between expectations and behaviors. Third, it identifies parental educational expectations as a “protective moderator,” challenging the simplistic view that high expectations are necessarily harmful. At the practical level, this study aims to remind parents and educators that frequent homework checking—a seemingly “responsible” behavior—may exacerbate adolescents’ loneliness through increased academic pressure, and that high expectations, when combined with supportive monitoring, can buffer this negative effect.

## 2. Theoretical Analysis and Hypotheses

To establish a solid theoretical foundation, it is necessary first to clarify the core constructs of this study. Parental homework checking frequency refers to how often parents actively review and supervise their children’s schoolwork completion, reflecting direct parental monitoring of children’s academic behavior (Pelham et al., 2024). Academic pressure denotes adolescents’ subjective perception of stress related to academic demands, including anxiety and worry about examinations, homework, and performance (Brata et al., 2025). Loneliness is an individual’s subjective experience of social isolation and emotional emptiness, serving as a key indicator of adolescent mental health (Verity et al., 2025). Parental educational expectations refer to the highest level of education that parents hope their children will ultimately attain, reflecting the family’s level of aspiration for children’s academic achievement (Rizwan et al., 2020). These constructs together form the theoretical framework of this study.

This study adopts the stimulus–organism–response (SOR) theory (Mehrabian & Russell, 1974) and the stress process model (Pearlin et al., 1981) as its overarching theoretical frameworks. The SOR framework provides a general architecture that delineates how external environmental stimuli (S) trigger internal psychological states (O), which in turn lead to behavioral or emotional responses (R). However, SOR alone does not specify the cognitive-evaluative mechanisms that translate a stimulus into a specific internal state. The stress process model fills this gap by explicitly theorizing the role of stress perception as the mediating link between stressors and adverse mental health outcomes. Integrating the two, we position parental homework checking as an external stimulus (S) and a chronic stressor, academic pressure as adolescents’ stress appraisal (O), and loneliness as the emotional response (R). This integrated framework directly supports our moderated mediation model. The following sections elaborate the relationships among the variables and present the corresponding research hypotheses.

### 2.1. The Relationship Between Parental Homework Checking Frequency and Academic Pressure

According to the SOR theory, frequent parental homework checking constitutes an external environmental stimulus (S). This stimulus has two notable features: (a) high frequency—homework checking is typically a daily, repetitive behavior; and (b) controlling nature—it signals “your performance needs to be monitored.” Together, these features determine its unique impact on adolescents’ psychological state.

From a cognitive appraisal perspective, frequent homework checking is accompanied by adolescents’ perception of a mandatory demand to ‘perform excellently.’ When such monitoring becomes overly frequent, adolescents interpret it as parental distrust or excessive intervention rather than supportive help. This appraisal triggers fear of academic failure and uncertainty about one’s own ability, thereby elevating academic pressure (Huang & Prochner, 2003). From the perspective of self-determination theory (Ryan & Deci, 2000), controlling parental behaviors undermine adolescents’ sense of autonomy and competence, and frustration of these two basic psychological needs is a core source of stress experiences (Soenens & Vansteenkiste, 2010). Specifically, when parents check homework frequently, adolescents feel that they cannot control their own learning process and are forced to comply with parental standards; this deprivation of autonomy translates into a state of sustained psychological tension.

Within the Chinese cultural context, the relationship between parental monitoring and psychological pressure has unique characteristics. Influenced by the Confucian tradition that “a successful scholar can become an official” and the value of filial piety, parental supervision is closely tied to “care” and “responsibility.” Consequently, adolescents find it difficult to directly refuse or express dissatisfaction with monitoring behaviors, because they fear that rejection would be seen as unfilial or as disappointing their parents’ expectations. This cultural constraint makes it easier for external pressure to be internalized as academic pressure: adolescents can neither change their parents’ monitoring behaviors nor escape anxiety about grades, leaving them to direct the pressure inward. Existing empirical research supports this reasoning. Using longitudinal data from the China Education Panel Survey, Lu et al. (2021) found that parental monitoring behaviors were positively associated with adolescents’ perceived academic pressure. Similarly, a recent systematic review and meta-analysis of 88 studies confirmed that maternal overprotection is uniquely associated with child anxiety symptoms, including academic anxiety (Manuele et al., 2023). Therefore, we propose:

**H1.**  *Parental homework checking frequency is positively associated with adolescents’ academic pressure.*

### 2.2. The Relationship Between Academic Pressure and Loneliness

As a chronic stressor, academic pressure has broad negative associations with adolescents’ mental health. According to the stress process model (Pearlin et al., 1981), sustained exposure to stress is related to an accumulation of psychological distress, and loneliness is a major manifestation of such distress (Goossens, 2018). Academic pressure may exacerbate loneliness through multiple pathways: high academic load crowds out time and energy for peer social interaction; persistent academic worry reduces social willingness and induces social avoidance; and negative self-evaluations stemming from academic pressure further undermine confidence in interpersonal interactions. Together, these mechanisms point to a core inference: the higher the academic pressure, the more likely adolescents are to feel lonely. Empirical research supports this relationship. A systematic review by Steare et al. (2023) found that academic pressure is one of the strongest predictors of adolescent emotional problems, including depression, anxiety, and loneliness. Deb et al. (2015) reported similar findings in a study of Indian high school students. Lan et al. (2023) directly validated a significant positive association between academic pressure and adolescent loneliness. Therefore, we propose:

**H2.**  *Academic pressure is positively associated with adolescents’ loneliness.*

### 2.3. The Mediating Role of Academic Pressure

Integrating the two pathways outlined above (H1 and H2), parental homework checking frequency may indirectly affect loneliness through academic pressure. This mediating mechanism can be explained from two theoretical perspectives.

From the SOR perspective, parental homework checking constitutes an external stimulus (S), academic pressure is the organism’s internal psychological state (O), and loneliness is the ultimate emotional-behavioral response (R). The core proposition of this theory is that external stimuli do not directly determine responses but rather exert indirect effects by altering the individual’s internal state. In the context of this study, parental homework checking is associated with loneliness precisely because this behavior is first linked to adolescents’ academic pressure—a cognitive-emotional state characterized by tension, anxiety, and loss of control. Without the intermediate link of academic pressure, monitoring behavior alone might not necessarily result in loneliness.

From the stress process model perspective, parental homework checking can be viewed as a “chronic stressor.” The model posits that stressors ultimately lead to adverse health outcomes such as psychological distress through the mediating mechanism of “stress perception” (Pearlin et al., 1981). Academic pressure is the core representation of stress perception; it reflects the individual’s cognitive appraisal and emotional reaction to the stressor. Only when adolescents perceive parental homework checking as stressful does this behavior further harm their mental health (manifested as loneliness). If adolescents view homework checking as normal or even helpful, then even high frequency may not produce negative consequences. Thus, academic pressure, as a mediator, captures the key link in the transformation process of “objective behavior → subjective perception → psychological outcome.”

Compared with more complex chain mediation models, a single-mediator model is more parsimonious and directly reflects the core pathway from external monitoring to psychological distress. Of course, parental homework checking may also affect loneliness through other pathways, such as undermining self-efficacy, reducing parent–child communication quality, or increasing parent–child conflict. However, as the most immediate emotional reaction, academic pressure should be the central mediating mechanism. Significance testing of the indirect effect will provide empirical evidence. Therefore, we propose:

**H3.**  *Academic pressure mediates the relationship between parental homework checking frequency and adolescents’ loneliness.*

### 2.4. The Moderating Role of Parental Educational Expectations

Parental educational expectations reflect parents’ aspirations for their children’s future academic achievement and constitute an important component of the family psychological environment (Seginer, 1983). According to an extended logic of the SOR theory, the strength of the relationship of external stimuli with an individual’s internal state may be moderated by contextual factors in the individual’s environment. Specifically, parental educational expectations may alter adolescents’ cognitive appraisals of homework checking behaviors, thereby moderating the “stimulus (S) → organism (O)” link.

Cognitive reappraisal mechanism under high expectations: When parents hold high expectations, adolescents are more likely to interpret parental homework checking as “my parents care about my future” and “my parents have confidence in me, so they are willing to invest time in supervising me,” rather than “my parents do not trust me.” This positive cognitive reappraisal weakens the sense of pressure induced by homework checking. At the same time, parents with high expectations tend to accompany homework checking with more emotional support and resource investment—they not only check homework but also patiently explain, provide learning materials, and offer encouraging feedback, rather than merely monitoring and controlling (Kim et al., 2015). This “supportive monitoring” satisfies adolescents’ needs for autonomy and competence, thereby buffering the potential pressure that monitoring itself may bring.

Pressure amplification mechanism under low expectations: In contrast, when parental educational expectations are low, adolescents tend to cognitively reappraise homework checking as a sign of distrust in their academic ability or as excessive control. Specifically, low expectations convey an implicit signal that “you cannot achieve higher academic success,” leading adolescents to interpret parental monitoring as a lack of confidence rather than supportive concern (Bai et al., 2025; Song et al., 2024). This negative cognitive appraisal further amplifies academic pressure. Moreover, parents with low expectations often lack emotional support when checking homework and are more likely to display impatience, blame, or negative evaluations, thereby reinforcing adolescents’ self-doubt and experience of pressure.

From the perspective of the stress-buffering hypothesis (S. Cohen & Wills, 1985), high expectations can serve as a psychological resource that buffers the impact of an external stressor (frequent homework checking) on stress perception. S. Cohen and Wills (1985) pointed out that resources such as social support exert their greatest protective effects under high-stress conditions—when stress is low, the buffering effect is not evident; when stress is high, the protective effect of resources becomes meaningfully stronger. In the present study, when parental homework checking frequency is very high, the buffering effect of high expectations is particularly salient; when homework checking frequency is low, the moderating role of expectations may be relatively weak.

Existing empirical research provides direct evidence for this moderating mechanism. Sakaki et al. (2026), using longitudinal data from German secondary school students, found that the interaction between parental educational expectations and parental monitoring behaviors significantly affected adolescents’ academic emotions—high expectations combined with supportive monitoring increased positive emotions and decreased negative emotions. Cross et al. (2019), in a study of Latino adolescents, found that parental educational expectations were positively associated with adolescent academic self-efficacy, and that this association was stronger among parents who transmitted fewer messages of shame or pressure. This suggests that the protective effect of parental educational expectations does not exist in isolation but depends on the emotional climate of family interaction—when parents express expectations with fewer negative evaluations and less pressure, the positive effects of expectations can be fully realized, thereby indirectly supporting the logic that expectations moderate the impact of monitoring behaviors. Therefore, we propose the following:

**H4.**  *Parental educational expectations negatively moderate the relationship between parental homework checking frequency and academic pressure.*

Specifically, the higher the parental educational expectations, the weaker the positive predictive effect of parental homework checking frequency on academic pressure, and consequently the smaller the indirect effect on loneliness via the mediating path.

### 2.5. Integrated Moderated Mediation Model

Integrating the above hypotheses, this study constructs a moderated mediation model. In this model, parental homework checking frequency affects loneliness through two pathways: a direct path (c′) and an indirect path via academic pressure (a × b). Parental educational expectations moderate the first half of the indirect path (the a path), i.e., the relationship between parental homework checking and academic pressure. Because the magnitude of the indirect effect depends on the strength of the a path, and the a path is itself moderated by parental educational expectations, the entire indirect effect varies across levels of expectations. Specifically, in the high-expectation group, the a path is weaker, and thus the indirect effect is smaller; in the low-expectation group, the a path is stronger, and thus the indirect effect is larger.

**Figure 1:**
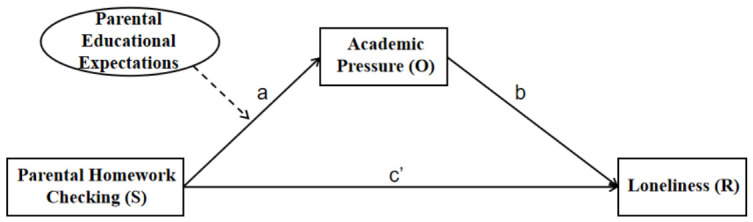
The moderated mediation model. Parental homework checking frequency (X) is related to adolescent loneliness (Y) both directly and indirectly through academic pressure (M). Parental educational expectations (W) moderate the relationship between X and M (the a path).

Based on the above theoretical analysis, this study constructs a moderated mediation model as shown in Figure 1.

## 3. Materials and Methods

### 3.1. Data Source and Sample

The data for this study were drawn from the 2022 wave of the China Family Panel Studies (CFPS). Conducted by the Institute of Social Science Survey (ISSS) at Peking University, the CFPS is a nationally representative, comprehensive longitudinal social survey covering 25 provinces, municipalities, and autonomous regions in China. Although the CFPS follows the same participants over multiple waves, the present study uses only the cross-sectional data from the 2022 wave because all key variables of interest were measured simultaneously in that wave and we focus on contemporaneous associations rather than change over time. The sampling design, data collection procedures, and quality control measures of the CFPS have been detailed in relevant technical reports (Xie & Hu, 2014).

This study used two types of data from the 2022 CFPS survey: (a) the child-reported parental questionnaire, which provided information on parents’ involvement in their children’s education, and (b) the individual self-report questionnaire, which provided adolescents’ self-reports on mental health, perceived pressure, and related measures. Data were merged using the unique personal identification code (pid) for one-to-one matching.

The sample inclusion criteria were as follows: (a) age below 18 years; and (b) presence in both the child-reported parental questionnaire and the individual self-report questionnaire, with no missing values on the core variables. The core variables included parental homework checking frequency, academic pressure, loneliness, parental educational expectations, age, and years of education. After applying these criteria, a final effective sample of 1831 adolescents was obtained.

Before exclusion, the initial merged dataset contained 2845 adolescents under age 18 with non-missing age and sex. After excluding 1014 cases with missing values on any of the core variables (parental homework checking, academic pressure, loneliness, parental educational expectations, age, or years of education), the final analytical sample was 1831. The majority of excluded cases (*n* = 997) were due to missing data on parental homework checking, which the CFPS child–parent questionnaire did not ask for children above age 15. Missing rates for other core variables were below 2%. Given that missingness was primarily due to the survey design (age restriction) rather than unobserved individual characteristics, listwise deletion is appropriate and unlikely to introduce bias.

The basic characteristics of the sample are presented in Table 1. Among the 1831 adolescents, 838 (45.77%) were female and 993 (54.23%) were male. Age ranged from 9 to 15 years; adolescents aged 16–17 were not present in the final sample because they had missing values on one or more core variables (primarily parental homework checking, which the CFPS child–parent questionnaire did not ask for children above age 15). The mean age was 12.35 years (SD = 1.71). The mean years of education was 6.08 (SD = 1.85), ranging from 1 to 13 years.

The CFPS employs a multistage probability-proportional-to-size sampling design covering 25 provincial regions. Sampling weights were not applied because our analyses focus on associational relationships rather than population parameter estimation.

To examine potential clustering, we calculated the intraclass correlation coefficient (ICC) of loneliness by urban–rural classification. The resulting ICC was 0.025 (95% CI [0.000, 0.096]), indicating that only 2.5% of the variance in loneliness is attributable to clustering. This negligible ICC justifies the use of single-level regression models without multilevel adjustments.

### 3.2. Variable Measurement

All variables were directly taken from the original items of the 2022 CFPS standardized questionnaire. Because the CFPS is a large-scale comprehensive social survey, each psychological construct was measured with a single item. This practice is widely accepted in secondary analysis studies based on large-scale social surveys (Mund et al., 2023; Cheung & Lucas, 2014). To ensure content validity, the selected items came from well-tested modules of the CFPS questionnaire that had undergone expert review and pretesting, and the wording of each item was highly consistent with the theoretical definition of the corresponding construct.

#### 3.2.1. Independent Variable: Parental Homework Checking Frequency

Parental homework checking frequency is an important indicator of the behavioral monitoring dimension of parental educational involvement, reflecting the extent of parents’ direct intervention in their children’s academic process (Grinshtain & Harpaz, 2021). This variable was derived from the child-reported parental questionnaire (item F604M). The original question asked: “How often do you check this child’s homework?” It was rated on a 5-point Likert scale: 1 = never, 2 = rarely (once a month), 3 = occasionally (once a week), 4 = often (2–4 times per week), and 5 = very often (5–7 times per week). Higher scores indicate more frequent parental homework checking. This item has clear behavioral specificity and has been used as a valid indicator of parental monitoring in multiple CFPS-based studies (J. Li et al., 2024; Gao et al., 2025). However, the measure captures only the frequency of checking, not its quality or emotional tone, which may be an important distinction for future research.

#### 3.2.2. Mediating Variable: Academic Pressure

Academic pressure refers to the psychological burden and tension an individual perceives when facing academic demands, and it is a core variable in research on academic emotions (Kumaraswamy, 2013). A large body of research has shown that excessive academic pressure negatively affects students’ mental health, academic performance, and overall well-being (Bedewy & Gabriel, 2015; Kaynak et al., 2023). In large-scale social surveys, single-item measures are often used to balance efficiency and the capture of core information. The data for this study came from the individual self-report questionnaire of the CFPS (item QS502). The original question asked: “How much pressure do you feel in your studies?” It was rated on a 5-point Likert scale: 1 = no pressure, 2 = a little pressure, 3 = moderate, 4 = relatively high pressure, and 5 = very high pressure. Higher scores indicate greater academic pressure. This single-item measure directly captures the overall subjective perception of academic pressure; it is conceptually consistent with classic perceived stress scales and has good face validity within the CFPS.

#### 3.2.3. Dependent Variable: Loneliness

Loneliness is a subjective emotional experience referring to an individual’s perceived social isolation or emotional alienation, and it is closely related to adolescent mental health (Twenge et al., 2021). This variable was derived from the individual self-report questionnaire (item QN414). The original question asked: “During the past week, how often did you feel lonely?” It was rated on a 4-point Likert scale: 1 = almost never (less than 1 day), 2 = sometimes (1–2 days), 3 = often (3–4 days), and 4 = most of the time (5–7 days). Higher scores indicate stronger feelings of loneliness. This item is adapted from the loneliness item of the Center for Epidemiologic Studies Depression Scale (CES-D) and has been shown to have good validity.

#### 3.2.4. Moderating Variable: Parental Educational Expectations

Parental educational expectations refer to parents’ psychological anticipation of the highest level of education their child will ultimately attain; they are a core predictor in educational sociology research on educational attainment and intergenerational transmission (Lai et al., 2022). A large number of studies have demonstrated that parental educational expectations are one of the most robust family factors predicting children’s academic achievement, and this effect remains significant even after controlling for family socioeconomic status (Jeynes, 2024). Moreover, parental educational expectations have a significant impact on adolescents’ academic motivation and mental health (Hu et al., 2024). This variable was derived from the child-reported parental questionnaire (item WD2). The original question asked: “What is the highest level of education you hope this child will complete?” The original response options were: 1 = illiterate/semi-literate, 2 = primary school, 3 = junior high school, 4 = senior high school/secondary vocational school/technical school/vocational high school, 5 = associate degree, 6 = bachelor’s degree, 7 = master’s degree, and 8 = doctoral degree. To facilitate continuous variable analysis, we converted these options into expected years of education: illiterate/semi-literate = 0 years, primary school = 6 years, junior high school = 9 years, senior high school = 12 years, associate degree = 14 years, bachelor’s degree = 16 years, master’s degree = 18 years, and doctoral degree = 21 years. The converted variable “expect_years” was entered as a continuous variable in the analysis. This conversion method is consistent with the coding of years of education commonly used in the economics of education and has been applied in many studies. Although this linear conversion assumes equal intervals between educational levels, it follows standard practice in educational economics and has been used extensively in CFPS-based research.

#### 3.2.5. Control Variables

We selected age and years of education as control variables. Age was calculated as a continuous variable by subtracting the birth year from 2022. Years of education was taken from the CFPS variable “cfps2022eduy,” which records the number of years of formal education the respondent had completed. The rationale for choosing these two variables is that age and years of education are closely related to adolescents’ cognitive development, stress coping ability, and experiences of loneliness; controlling for them helps reduce potential confounding bias.

Ideally, we would have controlled for additional confounders such as parent–child communication quality, family conflict, parental mental health, and adolescent personality traits. However, the CFPS 2022 dataset either did not include these variables for the same subsample or had excessive missing rates that would have compromised statistical power. Therefore, we limited control variables to age and years of education, which are consistently available and conceptually relevant to developmental and coping processes. We acknowledge this limitation and address it further in the discussion section.

### 3.3. Statistical Analysis

All statistical analyses were performed using Stata 17.0. The significance level was set at α = 0.05 (two-tailed). The analytical steps were as follows.

First, descriptive statistics and correlation analysis. We calculated the mean, standard deviation, and Pearson correlation coefficients for each variable to preliminarily examine the direction and strength of associations among the variables.

Second, common method bias test. Because all variables came from self-report data from the same survey, common method bias might exist. We conducted Harman’s single-factor test (Podsakoff et al., 2003) by performing an unrotated principal component factor analysis on the standardized scores of all core variables (parental homework checking frequency, academic pressure, loneliness, and parental educational expectations). The results are reported in Section 4.1.

Third, mediation effect test. We used the stepwise regression approach (He et al., 2025) combined with the bootstrap method (5000 resamples) to test the mediating role of academic pressure. The stepwise regression involved three steps: (a) testing the effect of the independent variable on the mediator (path a); (b) testing the effect of the mediator on the dependent variable (path b); and (c) testing the direct effect of the independent variable on the dependent variable after controlling for the mediator (path c′). The bootstrap method was used to compute the confidence interval for the indirect effect.

Fourth, moderated mediation effect test. Based on the mediation model, we added parental educational expectations and its interaction term with the independent variable to test the moderating effect. The bootstrap method (5000 resamples) was used to compute the index of moderated mediation and the confidence intervals for the conditional indirect effects. All continuous variables were standardized (mean = 0, SD = 1) before analysis to make the regression coefficients comparable.

Fifth, simple slope analysis. For significant interaction effects, we conducted simple slope tests to examine the effect of parental homework checking on academic pressure at low (mean − 1 SD) and high (mean + 1 SD) levels of parental educational expectations.

## 4. Results

### 4.1. Common Method Bias Test and Descriptive Statistics

Harman’s single-factor test was conducted on the four core variables (parental homework checking frequency, academic pressure, loneliness, and parental educational expectations) using an unrotated principal component factor analysis. The results showed that the first factor had an eigenvalue of 1.227 and explained 30.69% of the total variance, which is below the critical threshold of 40%. This indicates that common method bias is not a serious concern in this study, and further analyses can proceed. Although Harman’s single-factor test is the most widely used diagnostic for common method bias, it is known to have limited sensitivity and does not fully rule out the presence of common method variance (Podsakoff et al., 2003). More rigorous procedures, such as the unmeasured latent method factor technique, could not be applied in this study because each construct was measured with a single item, making a latent method factor unidentified. Thus, although the Harman test suggests no severe common method bias, this conclusion warrants caution.

The means, standard deviations, and Pearson correlation coefficients for the core variables are presented in Table 2. Parental homework checking frequency was significantly and positively correlated with academic pressure (r = 0.127, *p* < 0.001). Academic pressure was significantly and positively correlated with loneliness (r = 0.098, *p* < 0.001). The direct correlation between parental homework checking frequency and loneliness was negative and weak (r = −0.064, *p* < 0.01). Parental educational expectations showed a weak positive correlation with parental homework checking frequency (r = 0.136, *p* < 0.01), but were not significantly correlated with academic pressure (r = 0.033, *p* > 0.05) and showed a weak negative correlation with loneliness (r = −0.063, *p* < 0.01). These correlation patterns provide preliminary support for the hypothesized mediation and moderation effects.

### 4.2. Mediation Effect Test

The stepwise regression approach was used to test the mediating role of academic pressure between parental homework checking and loneliness. All models controlled for age and years of education. The results are presented in Table 3.

First, parental homework checking frequency was positively associated with academic pressure (B = 0.1596, SE = 0.0241, t = 6.63, *p* < 0.001), supporting H1. Second, academic pressure positively associated with loneliness (B = 0.0989, SE = 0.0237, t = 4.17, *p* < 0.001), supporting H2. Third, after controlling for the mediator, the direct effect of this behavior on loneliness was negative and significant (B = −0.0786, SE = 0.0249, t = −3.15, *p* = 0.002), indicating partial mediation.

The indirect effect of academic pressure (a × b) was 0.0176, with a 95% bootstrap confidence interval (5000 resamples) of [0.0028, 0.0329] (bias-corrected), which does not contain zero, indicating a significant mediation effect. Thus, H3 was supported. The indirect effect accounted for approximately 18.3% of the total effect, but the direct effect remained dominant, suggesting that other mediating pathways may exist. Although the indirect effect is modest in magnitude, it is statistically reliable and consistent with theoretical expectations, given the cross-sectional design and single-item measures.

### 4.3. Moderating Effect of Parental Educational Expectations

To test whether parental educational expectations moderate the relationship between parental homework checking and academic pressure, we added parental educational expectations and its interaction term with the independent variable to the mediation model. All continuous variables were standardized. The regression results are presented in Table 4.

The interaction term (parental homework checking × parental educational expectations) significantly and negatively associated with academic pressure (B = −0.1622, SE = 0.0162, t = −9.99, *p* < 0.001), indicating that parental educational expectations negatively moderated the relationship between parental homework checking and academic pressure. The index of moderated mediation (a_3_ × b) was −0.0179, with a 95% bootstrap confidence interval (5000 resamples) of [−0.0293, −0.0066], which does not contain zero, indicating a significant moderated mediation effect.

**Figure 2:**
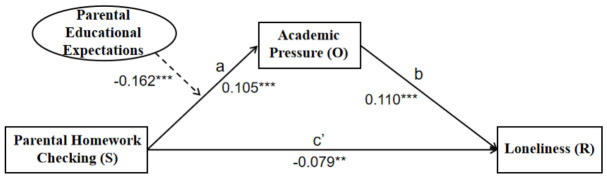
The moderated mediation model with standardized path coefficients. ** *p* < 0.01, *** *p* < 0.001.

To interpret the direction of the interaction effect, we divided parental educational expectations into high and low groups using the mean ± 1 SD method (J. Cohen et al., 2013). Specifically, the low-expectation group was defined as one standard deviation below the mean (W = −0.9987), corresponding to approximately 10.5 years of expected education (below senior high school). The high-expectation group was defined as one standard deviation above the mean (W = 0.1778), corresponding to approximately 16.5 years of expected education (bachelor’s degree or higher). Simple slope analysis (see Figure 2) showed that in the low-expectation group, the positive predictive effect of parental homework checking on academic pressure was stronger (B = 0.267, SE = 0.034, *p* < 0.001); in the high-expectation group, this effect was appreciably weaker (B = 0.076, SE = 0.029, *p* = 0.002). The *t*-test for the difference between the two slopes was 9.99 (*p* < 0.001). The conditional indirect effects showed that the indirect effect (the mediation effect of academic pressure between parental homework checking and loneliness) was 0.0295 (95% CI [0.0097, 0.0471]) in the low-expectation group and 0.0084 (95% CI [0.0003, 0.0244]) in the high-expectation group, with the former being approximately 3.5 times larger than the latter. Therefore, H4 was supported: the higher the parental educational expectations, the weaker the positive effect of parental homework checking on academic pressure, and consequently the smaller the indirect effect on loneliness via the mediating path.

To compare the moderated mediation model with a simpler mediation-only model (i.e., without the interaction term), we performed a likelihood-ratio test. The model with the interaction term showed a significantly better fit (LRX^2^(2) = 98.24, *p* < 0.001). The AIC decreased from 5090.59 to 4996.35, and the BIC decreased from 5112.64 to 5029.43, indicating that the moderated mediation model is preferable. These results support the inclusion of the moderating role of parental educational expectations.

To visually summarize the moderated mediation model and the magnitude of each path, we present Figure 2, which displays all standardized coefficients derived from the regression analyses reported in Table 3 and Table 4.

**Figure 3:**
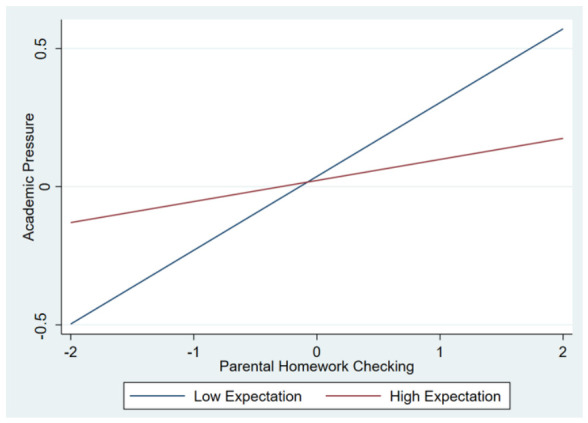
Moderating effect of parental educational expectations on the relationship between parental homework checking and academic pressure.

To further illustrate the direction and nature of the moderating effect of parental educational expectations, we conducted simple slope analyses. Figure 3 displays the simple slopes for the relationship between parental homework checking and academic pressure at low (mean − 1 SD) versus high (mean + 1 SD) levels of parental educational expectations.

## 5. Conclusions and Discussion

### 5.1. Conclusions

Drawing upon the stimulus–organism–response (SOR) theory and the stress process model, this study used large-sample data from the 2022 China Family Panel Studies (CFPS) to empirically examine the mechanism through which parental homework checking frequency affects adolescent loneliness, specifically investigating the mediating role of academic pressure and the moderating role of parental educational expectations. The main conclusions are as follows.

First, we found that parental homework checking frequency was positively associated with academic pressure, supporting H1. This finding supports the core proposition of the stress process model: frequent parental homework checking is perceived by adolescents as an external stressor rather than as supportive behavior. From the SOR perspective, parental homework checking constitutes an environmental stimulus (S), which adolescents interpret as monitoring or excessive intervention, thereby triggering an internal psychological state of tension—namely, academic pressure (O).

Second, academic pressure was positively associated with loneliness, supporting H2. Adolescents who experience higher academic pressure are more likely to feel social isolation and emotional loneliness. This result aligns with the stress-emotion model, extending the explanatory boundary of the stress process model by incorporating loneliness as a specific emotional outcome. From the SOR perspective, academic pressure (O) further triggers loneliness as a behavioral/emotional response (R), forming a complete chain of external monitoring → internal pressure → negative emotion.

Third, academic pressure partially mediated the relationship between parental homework checking frequency and loneliness, supporting H3. The indirect effect was statistically significant, and the direct effect remained significant, indicating partial mediation. This suggests that parental homework checking not only directly increases adolescent loneliness but also operates indirectly through the psychological pathway of academic pressure. The persistence of a significant direct effect implies that other unexamined mediators may exist, warranting further investigation.

Fourth, parental educational expectations significantly and negatively moderated the effect of parental homework checking on academic pressure, supporting H4. The interaction effect was significant, and the index of moderated mediation was also significant. Simple slope analysis showed that the positive association between homework checking and academic pressure was stronger in the low-expectation group and appreciably weaker in the high-expectation group. This moderating pattern is consistent with the stress-buffering hypothesis: high parental expectations acted as a psychological buffer rather than exacerbating negative outcomes. When parents hold high expectations, adolescents interpret those expectations as trust and investment, thereby perceiving homework checking as a sign of care and support rather than mere surveillance or control.

In summary, all four hypotheses were supported, forming a clear logical chain: frequent parental homework checking (S) is associated with adolescent loneliness (R) through academic pressure (O), and high levels of parental educational expectations weaken the negative association between homework checking and academic pressure. These findings validate the applicability of the SOR theory and the stress process model to the context of Chinese family education and provide clear targets for intervention—reducing mechanical homework checking, improving the way expectations are communicated, and transforming monitoring into support.

### 5.2. Theoretical Contributions

The theoretical contributions of this study are threefold.

First, this study extends the stimulus–organism–response (SOR) theory from consumer behavior and organizational management to the domain of family education and adolescent mental health, validating the explanatory chain of “external monitoring behavior → cognitive appraisal → emotional health outcome.” Previous applications of SOR theory have mostly focused on areas such as social media use (J. Li et al., 2024), online consumption (Sutanto & Utomo, 2025), workplace stress (Olfat, 2024), and educational technology acceptance (Y. Zhang et al., 2025). However, recent systematic reviews confirm that its application to parent–child interactions and specific educational behaviors remains exceptionally rare (Pelham et al., 2024). Using parental homework checking frequency as the external stimulus (S), academic pressure as the organism’s cognitive-emotional state (O), and loneliness as the ultimate behavioral/emotional response (R), this study constructed a three-stage path model. Compared with complex chain mediation models, the single-mediator model (with academic pressure as the sole mediator) was statistically significant and structurally more parsimonious, suggesting that academic pressure is a key cognitive-evaluative variable linking parental monitoring behavior to adolescent loneliness. This finding confirms the applicability of SOR theory to family education contexts. It also echoes the core logic of the stress process model: “stressor → stress perception → adverse outcome” (Pearlin et al., 1981).

Second, this study distinguishes between the “behavioral” dimension (homework checking frequency) and the “expectational” dimension (educational expectation level) of parental educational involvement, revealing the psychological costs of specific monitoring behaviors and the interactive mechanism between expectations and behavior. Existing research often treats parental educational involvement as a global construct or examines the effects of educational expectations or monitoring behaviors separately (Pinquart & Ebeling, 2020; Pelham et al., 2024), rarely examining both their independent and interactive effects within the same model. The present study found that the specific behavior of homework checking significantly associated with academic pressure, and this effect was significantly negatively moderated by parental educational expectations. In other words, high expectations do not invariably amplify the negative associations of monitoring—when expectations are sufficiently high, they can actually weaken the pressure induced by homework checking. This finding challenges a simplistic “monitoring is harmful, expectations are also harmful” dichotomy and suggests that researchers should attend to the match between behavior and expectations. Theoretically, this aligns with the distinction in self-determination theory (Ryan & Deci, 2024) between “autonomy support” versus “control”: when high expectations are communicated in a supportive manner (e.g., “I believe you can do it”), they can satisfy children’s needs for competence and autonomy; low expectations, in contrast, make it more likely that children will interpret monitoring behavior as distrust.

Third, this study reveals the role of parental educational expectations as a “protective moderator,” challenging the popular view that high expectations necessarily increase pressure. For a long time, high parental expectations have often been associated with negative outcomes such as academic anxiety, perfectionism, and psychological burnout (Curran & Hill, 2019). However, the moderation analysis in this study found that under conditions of high-frequency monitoring, the high-expectation group reported appreciably lower academic pressure than the low-expectation group. Simple slope analysis showed that the positive association between homework checking and academic pressure was stronger in the low-expectation group and appreciably weaker in the high-expectation group. This result supports the core hypothesis of the stress-buffering model (Landerman et al., 1989)—that a resource (here, high expectations) can buffer the relationship between a stressor (here, frequent homework checking) and the stress response. From a cognitive appraisal perspective, high expectations may alter adolescents’ interpretive framework for parental monitoring behavior: when children believe their parents have high expectations for them, they are more likely to view homework checking as “concern for my future” rather than “monitoring my shortcomings.” This finding not only enriches our understanding of expectation effects in parent–child interactions but also points to new directions for future research—for example, exploring how the manner in which expectations are expressed (e.g., warm and supportive vs. harsh and controlling) moderates their impact.

While we interpret the negative moderating effect of parental educational expectations as a “buffering” mechanism that weakens the positive association between homework checking and academic pressure, alternative explanations must be considered. First, parents who hold high educational expectations may differ systematically in other parenting characteristics—such as providing more emotional warmth, offering greater learning resources, or engaging in less controlling monitoring—which could independently reduce adolescents’ academic pressure. Prior research has shown that parental warmth and autonomy support are powerful predictors of adolescent well-being (Soenens & Vansteenkiste, 2010), and these qualities may co-occur with high expectations. Thus, the observed buffering effect might be partly attributable to these correlated parenting practices rather than to expectations per se. Second, adolescents in high-expectation families may have higher academic self-efficacy or stronger intrinsic motivation, both of which could mitigate the stressful interpretation of homework checking (Bandura, 1997; Ryan & Deci, 2024). Third, it is possible that a third variable, such as family socioeconomic status or parent–child communication quality, simultaneously is associated with higher expectations and lower pressure, producing a spurious moderation (Sirin, 2005). Because our data lack fine-grained measures of parenting styles or motivational constructs, we cannot rule out these alternative explanations. Future research should disentangle these mechanisms using longitudinal designs and more comprehensive assessments of family interaction, ideally including observational measures of parent–child dynamics.

In summary, this study makes theoretical contributions by transferring SOR theory to a novel context, distinguishing between behavioral and expectational dimensions of educational involvement, and providing the first empirical demonstration of the buffering effect of expectations. These contributions offer a new analytical framework for understanding the double-edged sword effect of Chinese family education.

### 5.3. Practical Implications

Given the cross-sectional and associational nature of our findings, the following implications are tentative and suggestive rather than prescriptive. They are intended to generate hypotheses for future longitudinal and intervention research, not to provide definitive guidelines.

For parents, the first step is to recognize the potential psychological costs of frequent homework checking, while not blindly lowering their educational expectations. We found that parental homework checking frequency is indirectly associated with adolescent loneliness through academic pressure, and that higher educational expectations actually buffer this negative effect. Based on this, parents may wish to adopt “supportive monitoring” instead of “controlling monitoring”: use encouraging language when checking homework, focus on the learning process rather than solely on grades or mistakes, give children space to arrange their own homework, and provide necessary learning resources rather than simply exerting pressure. Parents can also work with their children to set learning goals, translating high expectations into concrete, achievable incentives rather than abstract psychological burdens, thereby reducing the pressure associated with monitoring while maintaining expectations.

For schools, stress management could be incorporated as a core component of mental health education. The results indicate that academic pressure is an important antecedent of loneliness, suggesting that schools might consider helping students develop healthy stress coping strategies. Specifically, schools could offer “stress cognitive reappraisal” courses to teach students to identify and challenge irrational academic beliefs, such as automatic negative thoughts like “my parents checking my homework means they don’t trust me.” At the same time, schools may use parent education sessions to disseminate scientific approaches to educational involvement, helping parents understand that high expectations are not inherently harmful—the key lies in whether expectations are combined with supportive behaviors. Only when expectations are expressed in a warm, encouraging, rather than critical or controlling, manner can they exert a positive buffering effect.

For policymakers, attention could be given to the dissemination and standardization of family education guidance services. Community-based and school-based institutions may offer workshops on “positive discipline” that systematically cover stress identification, emotional communication skills, parent–child boundary setting, and appropriate ways to express expectations. Through such intervention programs, parents might be helped to shift from “excessive monitoring” to “moderate support,” thereby preventing the accumulation of academic pressure and the emergence of loneliness at the family level, addressing current gaps in the practical implementation of family education guidance services.

### 5.4. Limitations and Future Directions

Although this study reveals important relationships among parental homework checking, academic pressure, loneliness, and educational expectations using large-sample data, several limitations should be acknowledged and addressed in future research.

First, the cross-sectional design precludes causal inference. The CFPS 2022 data are from a single time point. Although the theoretical model posits parental homework checking as the independent variable, academic pressure as the mediator, and loneliness as the outcome, the possibility of reverse causality or bidirectional relationships cannot be completely ruled out. For example, lonely adolescents may exhibit more academic problems, which could in turn induce more frequent homework checking by parents. Future research should employ longitudinal designs with cross-lagged panel models to examine temporal ordering. Alternatively, quasi-experimental designs could strengthen causal inference.

Second, the set of control variables was limited. In this study, we controlled only for age and years of education. Unmeasured confounding factors—particularly parent–child relationship quality, family emotional climate, parental mental health, and adolescent personality traits—may influence both parental homework checking and adolescent loneliness. If these omitted variables are correlated with the independent variable and also independently predict loneliness, our estimates could be biased. Due to data constraints in the CFPS 2022 (i.e., these variables were either not available for the same subsample or had excessive missing rates), we could not include them. Future research should incorporate a richer set of covariates, ideally measured prospectively, to reduce omitted variable bias.

Third, the reliance on single-item measures may introduce reliability limitations. In the CFPS database, parental homework checking frequency, academic pressure, loneliness, and parental educational expectations were each measured with a single item, precluding the calculation of internal consistency reliability (Cronbach’s α) and making the measures susceptible to random measurement error. Although single-item measures have the advantage of brevity in large-scale social surveys and have been shown to have acceptable validity for certain constructs (Robins et al., 2001), future research is encouraged to replicate the findings using established multi-item scales to enhance the reliability and stability of the results.

Fourth, the sample characteristics and cultural context limit generalizability. This study focused on Chinese adolescents aged 9–15; the findings may not extend to older adolescents who face heavier academic burdens and stronger autonomy needs. Moreover, the conclusions are based on a Chinese family sample embedded in a Confucian heritage culture that emphasizes academic achievement and filial responsibility. In other cultural contexts (e.g., individualistic societies where parental monitoring is less intensive), the strength or even direction of the relationships may differ. Future research should include broader age ranges and cross-cultural samples to test the generalizability of the model.

Fifth, our assessment of common method bias relied solely on Harman’s single-factor test, which is a relatively insensitive diagnostic. Although the test indicated no severe bias, this procedure has been criticized for its low power to detect method effects, especially when measures are single-item (Podsakoff et al., 2003). Future research should employ multi-item scales and more rigorous approaches to more definitively rule out common method variance.

Sixth, the stepwise regression approach may have inflated Type I error. While convenient for exploratory variable selection, stepwise regression is prone to capitalizing on sampling error. We did not apply structural equation modeling (SEM) or PROCESS, which offer global fit indices, direct tests of indirect effects, and the capacity to model nested structures. Future studies should therefore employ SEM (or PROCESS) with multi-item scales to validate and extend our findings.

Despite these limitations, this study systematically reveals, through large-sample empirical analysis, the mechanism by which parental homework checking frequency affects adolescent loneliness through academic pressure, and identifies the negative moderating role of parental educational expectations. These findings not only provide a new perspective for understanding the double-edged sword effect of Chinese family education but also lay an empirical foundation for subsequent theoretical integration and intervention design.

## Figures and Tables

**Table 1:** Sample characteristics (*N* = 1831).

Variable	Category/Indicator	Value
Age (years), Mean ± SD		12.35 ± 1.71
Age range		9–15
Sex, n (%)	Female	838 (45.77%)
	Male	993 (54.23%)
Years of education, Mean ± SD		6.08 ± 1.85

**Table 2:** Descriptive statistics and correlation matrix (N = 1831).

Variable	M	SD	1	2	3	4
1. Parental homework checking	0.01	0.98	1			
2. Academic pressure	0.00	0.98	0.127 ***	1		
3. Loneliness	0.00	1.00	−0.064 **	0.098 ***	1	
4. Parental educational expectations	0.00	1.00	0.136 ***	0.033	−0.063 ***	1

Note: M = mean; SD = standard deviation. All variables were standardized (M = 0, SD = 1). ** *p* < 0.01, *** *p* < 0.001 (two-tailed).

**Table 3:** Mediation regression results (*N* = 1831).

Path	Predictor → Outcome	B	SE	t	*p*	95% CI
a	Parental homework checking→ Academic pressure	0.1596	0.0241	6.63	<0.001	[0.1124, 0.2069]
b	Academic pressure→Loneliness	0.0989	0.0237	4.17	<0.001	[0.0524, 0.1454]
c′	Parental homework checking→Loneliness	−0.0786	0.0249	−3.15	0.002	[−0.1275, −0.0297]
Indirect effect	a × b	0.0176	0.0076	(Bootstrap)	0.020	[0.0028, 0.0329]

Note: All models controlled for age and years of education. Bootstrap confidence intervals are based on 5000 resamples (percentile).

**Table 4:** Moderated mediation regression results (standardized coefficients, *N* = 1831).

Dependent Variable	Predictor	B	SE	t	*p*	95% CI
Academic pressure (M)	Parental homework checking (X)	0.1050	0.0242	4.34	<0.001	[0.0576, 0.1524]
	Parental educational expectations (W)	−0.0124	0.0226	−0.55	0.583	[−0.0567, 0.0319]
	X × W	−0.1622	0.0162	−9.99	<0.001	[−0.1941, −0.1304]
Loneliness (Y)	Academic pressure (M)	0.1105	0.0239	4.62	<0.001	[0.0635, 0.1574]
Index of moderated mediation		−0.0179	0.0058	(Bootstrap)	0.002	[−0.0293, −0.0066]
Conditional indirect effects	Low expectation (W = −0.9987)	0.0295				[0.0097, 0.0471] (P)
	High expectation (W = 0.1778)	0.0084				[0.0003, 0.0244] (BC)

Note: All models controlled for age and years of education. Bootstrap confidence intervals were based on 5000 resamples. P = percentile; BC = bias-corrected.

## Data Availability

The data that support the findings of this study are available from the China Family Panel Studies (CFPS). Restrictions apply to the availability of these data, which were used under license for this study, and they are not publicly available because Peking University, the data owner, does not permit secondary distribution of CFPS data on journal websites or any other third-party platforms. Data are available from the corresponding author upon reasonable request, subject to the CFPS data use agreement. The CFPS public-use dataset can also be directly accessed by qualified researchers through the official data portal at https://www.isss.pku.edu.cn/cfps/ (accessed on 20 May 2026). The analysis code used in this study is available from the first author upon reasonable request.
